# “I'm Not Here to Teach You How to Be Gay:” LGBTQ+ Client Experiences of Othering in Emotionally Focused Couple Therapy

**DOI:** 10.1111/jmft.70141

**Published:** 2026-05-06

**Authors:** Caitlin Edwards, Lekie Dwanyen, Andrea Wittenborn, Robert Allan

**Affiliations:** ^1^ Human Development and Family Studies Michigan State University East Lansing Michigan USA

**Keywords:** Emotionally Focused Couple Therapy, LGBTQ+, othering

## Abstract

Despite the increasing emphasis on addressing the intersections of client and therapist identity to improve therapeutic outcomes, therapy clients holding marginalized identities continue to experience othering. Othering is a process that engenders marginalization and inequality based on preconceived group identity, involves the hegemonic construction of belonging and not belonging, and often entails both stereotyping and racialization. This paper reports on the experience of lesbian, gay, bisexual, transgender, queer/questioning plus (LGBTQ+) emotionally focused couple therapy (EFCT) clients' experience of othering both in their own therapy and as part of participating in a research study on tailoring EFCT to account for the unique lived experience of members of the LGBTQ+ communities. This study relied on negative case analysis in the context of thematic analysis (TA) to develop three themes: (1) understanding versus inflating identity, (2) educating the therapist, and (3) deepening discussions around identity.

Extant research documents the positive outcomes of culturally responsive psychotherapy, including couple therapy (Ellis et al. 2022; Hall et al. 2019; Soto et al. 2018). An inherent aspect of culturally responsive therapy is the integration of client and therapist identities (Davis et al. 2018; Pettyjohn et al. 2020). Despite research supporting a multicultural orientation to therapeutic work (e.g., Jennings and Sprankle 2023; Owen et al. 2014), clients who hold marginalized identities continue to experience othering in therapy. Othering is a multidimensional process by which a dominant in‐group focuses on the “lacking“ characteristics of an outgroup in a reductionist way, leading to discrimination and marginalization (Jensen 2011). To better understand this persistent gap between the promise of culturally responsive practice and the realities clients face, this paper reports on Lesbian, Gay, Bisexual, Transgender, Queer/Questioning plus (LGBTQ+) Emotionally Focused Couple Therapy (EFCT) client experiences of being othered—both within their own therapy, and in the context of participating in a research study (Edwards et al. 2025).

## 1 Therapist and Client Identity

Both therapists and clients bring their own distinct identities into therapy; these identities interact throughout the course of therapy, thereby creating a blend of potentially concordant perspectives, cultures, and experiences (Pettyjohn et al. 2020). Client and therapist identity, for example, impacts therapeutic alliance (i.e., a collaborative relationship with consensus around therapy goals and tasks [Cruwys et al. 2023]). Shared social identity predicts both working alliance and the client's perceptions of the usefulness of therapy (Cruwys et al. 2023), while therapist competence navigating racial and ethnic differences depends on both client and therapist gender (Kivlighan et al. 2019); therapists appear to miss the intersections of oppression experienced by women of color (Kivlighan et al. 2019). Identity also impacts client perception of therapist competency: racial and ethnic minority clients experience racial/ethnic minority therapists as more authentic and culturally competent (Phillip and Maimon 2023).

Furthermore, holding a marginalized identity impacts therapy utilization, as people who hold historically marginalized identities are more reluctant to enter therapy (Jones 2024) and racial/ethnic minority clients drop out of therapy earlier (Zeber et al. 2017). Therefore, therapists working with clients who hold different identities than themselves (e.g., cisgender therapists working with transgender clients) may experience unique challenges, have unanticipated learning experiences, and need to develop essential knowledge and skills that support their ability to engage in effective therapeutic work (McGeorge et al. 2021; Salpietro et al. 2019). One aspect of the knowledge and skills needed to navigate the complexity of therapist and client identity in therapy is the ability to conceptualize how identities interact.

### 1.1 Models and Frameworks

Several frameworks can help therapists conceptualize interactions between therapist and client identities, including Intersectionality (Crenshaw 1989), Multicultural Orientation (MCO; Davis et al. 2018; Owen 2013; Owen et al. 2011), and Hays (2024) ADDRESSING model. Intersectionality (Crenshaw 1989) emphasizes lived experiences as multifaceted, dynamic, and subjectively influenced by persons' social locations; that is, their proximity to structural advantages and disadvantages based on their intersecting identities. This framework also recognizes proximity to systemic marginalization (e.g., poverty, caregiver incarceration, forced family separation) as core to social location and expands beyond individual identity. An intersectional lens illuminates how perceived group membership exposes individuals and relationships to unique and complex forms of discrimination and bias (Crenshaw 1989). Because an individual's own understanding of their identities is unique, their experience of bias and discrimination will also inherently be unique (Crenshaw 1989).

The Multicultural Orientation framework (MCO; Davis et al. 2018; Owen 2013) proposes ways of *being with* clients. The three MCO pillars include cultural humility, cultural comfort, and cultural opportunities. Cultural humility reflects a therapist's ability to establish egalitarian relationships with clients while remaining self‐aware and non‐defensive regarding their own ability to be other‐oriented. Cultural comfort is therapists' level of genuine comfort holding multicultural conversations in therapy, and cultural opportunities reflect therapists' actions to explore cultural identity (Davis et al. 2018; Owen 2013). Research shows that therapists with high levels of cultural humility and cultural comfort—who also act on cultural opportunities—have better therapeutic outcomes (Davis et al. 2018; Owen et al. 2016).

The ADDRESSING model highlights cultural norms and systems that grant or deny privilege and allows for the understanding of in‐group diversity (Hays 2024). The acronym can help therapists evaluate how their identities may influence the therapeutic relationship and how clients may experience the world based on their intersecting identities: Age/generation, Developmental/other Disability, Religion/spirituality, Ethnicity/racial identity, SES, Sexual orientation, Indigenous heritage, National origin, and Gender (Hays 2024). ADDRESSING encourages therapists to (1) conduct ongoing self‐assessments about how identity shapes therapeutic work, (2) attune to structural inequities embedded in the mental health system, (3) address systemic oppression, and (4) focus on client strengths (Hays 2024). Although numerous models and frameworks help therapists to conceptualize and address how client–therapist identities interact throughout therapy, many therapists may miss cultural opportunities (Davis et al. 2018), or inadvertently, say or do something that others their client.

## 2 Othering

Generally, othering is a process that engenders marginalization and inequality based on preconceived group identity (Powell and Menendian 2016) and involves the hegemonic construction of belonging and not belonging (Akbulut and Razum 2022). Othering occurs when an individual or group is systematically or interpersonally ascribed traits that are experienced as foreign, undesirable, or alien (Milkavcic and LeBlanc 2014) and often entails both stereotyping and racialization (Powell and Menendian 2016). To engage in othering, an in‐group must understand identity as dichotomous, thereby creating an out group (Akbulut and Razum 2022). Indeed, othering presupposes that society consists of real, distinguishable groups, and, thus, assumes that genuine group differences exist (Akbulut and Razum 2022).

### 2.1 Othering in Psychotherapy

Unfortunately, othering occurs in therapy, as the ways in which power and oppression exist in the therapy room reflect the ways these dynamics play out in the larger sociocultural context (Moodley 2009). Therapists who invalidate and de‐center marginalized voices outside the therapy room can enact these processes with their clients, while therapists who emphatically attune to clients' experiences of oppression and marginalization are likely to do so outside of therapy (Kuo et al. 2022). The act of othering by a therapist negatively impacts access to quality treatment and, therefore, exacerbates existing health disparities (Bhugra et al. 2023).

Othering is often a complex process depending on both the persons involved and the context (Powell and Menendian 2016). There may be parts of an individual that are othered (e.g., ability status), while other aspects of identity are embraced (e.g., race). Indeed, othering occurs when an undesired aspect of social identity is visible, but also contextually devalued (Powell and Menendian 2016). Othering may also be done unintentionally or enacted out of ignorance by therapists who are unaware of how identity impacts therapeutic work. Thus, the use of the models and frameworks for understanding how client and therapist identity interact are necessary to limit clients' experiences of being othered.

### 2.2 Microaggressions

Microaggressions (i.e., common, brief interpersonal experiences that send denigrating messages about a group of people based on unconscious biases and beliefs [Sue et al. 2022]) are a common form of othering. Microaggressions can be verbal or non‐verbal, are often indirect, can occur in situations in which there are alternative explanations for a trait or behavior, and are more likely to occur when people pretend not to notice differences (Sue et al. 2022). Those who perpetuate microaggressions often take an individualistic view of people's traits and behaviors, thereby denying the systematic, cognitive, and behavioral impact of race, ethnicity, gender, sexual orientation, ability status, and religion/spirituality (Sue et al. 2022). There are several types of microaggressions, including microinsults (subtle putdowns), microassaults (overt offenses), and microinvalidations (i.e., the denial of an individual's experience, especially of marginalization and oppression [Sue et al. 2022]). When microaggressions occur in therapy, they are associated with worse client outcomes (Davis et al. 2016). For example, transgender clients report a lack of respect for their gender identities (e.g., misgendering), the conflation of sexual and gender identity, and an overemphasis on gender dysphoria as a diagnosis, which negatively impacts therapy outcomes (Morris et al. 2020).

## 3 Current Study

The current paper is part of a larger study designed to understand LGBTQ+ clients' experiences engaging in EFCT and their recommendations for adapting EFCT. As part of engaging in this process, participants discussed identity‐affirming experiences in therapy as well as experiences that felt othering. This paper describes participants' reports of feeling othered in both their own therapy and as part of engaging in this study.

### 3.1 EFCT

EFCT (Johnson 2019) is a well‐researched and evidence‐based approach to relational treatment (Spengler et al. 2022). EFCT integrates elements of systems theory, experiential methods, and humanistic family therapy and is grounded in attachment theory (Johnson 2019). The interventions associated with EFCT aim to foster greater emotional attunement between partners, reduce destructive interaction patterns, and strengthen attachment bonds (Johnson 2019).

#### 3.1.1 EFCT With Diverse Populations

The empirical literature describing EFT applied to diverse populations is limited. “Pure” EFCT has been studied with Iranian couples (e.g., Ahmadi et al. 2014; Najafi et al. 2015), Taiwanese couples (Tseng et al. 2024), and heterosexual African American couples (Nightingale 2019). Yet, both conceptual (e.g., Allan and Johnson 2016; Guillory 2021) and empirical articles have begun to emerge to address these gaps. For example, broad cultural adaptations of EFCT have been described by Allan et al. (2022), Hattori (2014) illustrates a culturally adapted EFCT with Japanese couples, and Edwards et al. (2025) document EFCT therapist recommendations for the use of EFCT with LGBTQ+ individuals and relationships.

## 4 Methods

### 4.1 Study Design

This study was originally designed to gather information from EFCT clients who self‐identified as a member of the LGBTQ+ communities regarding how to tailor EFCT to account for their unique lived experiences. These core findings are described in Edwards et al. (2025). This paper specifically focuses on the unanticipated theme of othering, which emerged both as participants talked about their personal experiences of EFCT as well as when they engaged in the study process—theater testing.

#### 4.1.1 Theater Testing Focus Groups

Theater testing is a method of data collection that developed within market research to obtain participants' feedback on products, services, and advertisements (National Cancer Institute 2004). Theater testing involves a small group of targeted participants interacting with and/or discussing audio‐visual materials (National Cancer Institute 2004). In market research, theater testing is often combined with focus groups (National Cancer Institute 2004). The use of theater testing focus groups allows for gathering a range of opinions and experiences and understanding *why* focus group participants think and feel the way they do about the audiovisual material (Hodock 1980; Whitehill King et al. 1993). In market research, this provides researchers the opportunity to make changes to better sell the product or service (Atkin and Freimuth 2013).

When used in intervention research, theater testing using focus groups involves the intervention's target audience observing and discussing the intervention's impact, feasibility, and the potential for culturally relevant changes (Wingood and DiClemente 2008). Specifically, theater testing can be used to gather the first‐person voices and experiences of particular cultural groups to determine how to increase the cultural relevancy of an intervention (Wingood and DiClemente 2008). Cultural adaptations that have emerged from the use of theater testing include an increased focus on self‐management of HIV symptoms in Thailand (Khumsaen and Stephenson 2017) and the development of and HIV prevention app for men who have sex with men (MSM; Goldenberg et al. 2015).

### 4.2 Recruitment and Participants

The study was advertised via the queer EFCT listserv, the International Centre for Excellence in Emotionally Focused Therapy (ICEEFT) listserv, and queer EFCT Facebook groups between April and October of 2023. EFCT center leaders and community organizers were also contacted and asked to share information about the study with their local EFCT networks. All therapists in English‐speaking countries listed in the ICEEFT directory received email invitations. Advertisements included details about the study's purpose, procedures, voluntary nature, and compensation.

To be eligible, individuals had to: (1) self‐identify as a member of the LGBTQ+ communities, (2) have completed a minimum of six EFCT sessions with a partner they had been in a relationship with for at least 1 year, and (3) be 18 years of age or older. Participants were excluded if they were experiencing active psychosis, intimate partner violence, or suicidal ideation. Those who reported suicidal ideation during screening were referred to the 988‐crisis line and relevant local support resources.

A total of 35 LGBTQ+ identifying individuals participated. Most participants identified as White (71%) and were from the United States (89%). The rest resided in Belgium (3.67%), Canada (3.76%), and Australia (3.76%). Two participants identified as multiracial, two as Asian, two as Black, and two chose not to specify their race. Three participants identified as Hispanic, Latino/a/x, or of Spanish origin—two identified as Mexican, Mexican American, or Chicana/o/x, and one identified as Brazilian. Seven participants reported they worked as therapists, four of whom specifically had training in EFCT, and six of whom held master's degrees, while one held a doctoral degree. Most participants (95%) expressed satisfaction with EFCT. Other demographic information (e.g., age, annual income) has been reported in Edwards et al. (2025).

### 4.3 Data Collection Procedures

Data collection began following Michigan State University IRB (#8915) approval. One week before each focus group session, participants received an email containing the agenda and objectives, the interview guide, a Microsoft Teams meeting link, and links to the consent form and demographic questionnaire. A follow‐up reminder email was sent 24 h before the scheduled session. The focus groups ranged in length from 105 to 142 min, with an average duration of 122 min (see Edwards et al. (2025) for further details regarding methodology).

In line with Krueger and Casey's (2015) recommendations for conducting focus groups, the focus groups began with a series of opening questions, focused on how participants found their EFCT therapist and how their therapist fostered a sense of safety. Participants then watched two segments from the EFCT training videos *The EFT Path to Secure Connection: Working Successfully with Same‐Sex Couples* (Reel Concepts for Susan Johnson Inc. 2011). The first clip focused on identifying negative interaction cycles with a gay male couple, while the second showcased re‐engaging a withdrawn partner with a lesbian couple. Both sessions featured the same expert EFCT therapist, trainer, and supervisor demonstrating EFCT to fidelity. These videos illustrated standard EFCT practices but did not incorporate sociocultural strategies commonly used with marginalized populations. Therefore, while the videos modeled effective and “pure” EFCT, there was no explicit goal to reflect culturally responsive and/or affirmative therapy practices.

### 4.4 Data Analysis

Focus group transcripts were generated by Microsoft Teams and used for data analysis thereafter. Prior to analysis, the first author reviewed each focus group transcript while listening to corresponding audio recordings and correcting any transcription errors. Flexible and inductive analytic procedures were employed to account for richness that emerged *within* the data. All data were analyzed using TA procedures, a six‐step method for analyzing qualitative data. The six steps (Braun and Clarke 2006) include (1) becoming familiar with the data, (2) generating initial codes, (3) identifying potential themes, (4) reviewing those themes, (5) defining and naming the themes, and (6) producing the final report.

To begin familiarization, the first author listened to each focus group recording while taking notes and then read through each transcript four times, as recommended to enable deep engagement with the data (Saldana 2021). Notetaking also helped this process by documenting key takeaways from each group and initial impressions of the data. Next, initial codes were generated by identifying key words, concepts, brief phrases, and in vivo codes (participants' exact words) that were essential to key research questions.

Next, to identify potential themes, the first author began by identifying patterns, recurring ideas, and potential categories that could organize key codes. This was followed by latent coding, which is used to interpret deeper, underlying meanings and concepts based on the context of the data (Saldana 2021). The latent codes were then indexed using a standardized procedure in Microsoft Word. During coding, several salient themes emerged that were not explicitly anticipated in the original study design. These themes were incorporated throughout subsequent rounds of analysis and theme generation through an iterative process of code refinement and memoing. The emergence of these findings prompted frequent reflexive consideration regarding the original purpose of the study, as unanticipated findings can occur during qualitative research (Creswell and Poth 2018).

Therefore, internal and external audits were conducted by the second, third, and fourth authors to strengthen the trustworthiness of the study. The third author conducted internal audits of the data trail, which ensured alignment between existing data (audio recordings, transcripts, first author notes), and final themes and subthemes. All authors conducted external audits to assess the final representation of the results aligned with the study goals, methods, and participant statements. This ensured that codes, themes, and the final report stayed as close to participant perspectives as possible, especially the unanticipated, emergent theme of othering. These processes supported researcher reflexivity and helped to manage potential assumptions and biases throughout the research process.

#### 4.4.1 Trustworthiness

The credibility of qualitative research is evaluated based on how clearly the data and research methods are presented (Hadi and José Closs 2016). To enhance transparency in this study, the authors provided a detailed description of the research procedures and evidence of triangulation (i.e., using multiple data sources to develop a well‐rounded understanding of the topic [Hadi and José Closs 2016]), including notes and memos developed by the focus group co‐facilitator. To verify the accuracy of the transcripts, each participant was emailed a copy of their responses for review (i.e., member checking). Member checking allows participants to confirm whether their perspectives have been accurately represented, thus helping the researcher ensure authenticity in reporting their experiences (Creswell and Poth 2018). Three participants reviewed their transcripts and confirmed that their views were accurately reflected, while two other participants shared notes they had taken during the focus group sessions.

At the start of this project, the authors did not expect to find that the discussion of identity would result in participants feeling othered; extant research strongly supports the integration of identity into the therapeutic process, especially for LGBTQ+ clients (Neff 2024). Therefore, this paper focuses on negative case analysis, which involves the deliberate analysis and inclusion of data that does not fit theoretical predictions or expected patterns (Hanson 2017; Miles et al. 2019). Negative case analysis enhances the trustworthiness and rigor of qualitative research, as it involves a full investigation into factors that could explain the unexpected data and allows researchers to revise theoretical assumptions (Hanson 2017; Miles et al. 2019).

#### 4.4.2 Author Positionality

The first author, a White, pansexual, polyamorous, agender, femme‐presenting individual from a lower‐middle‐class background, maintained reflective journals throughout the project to examine how their social identities shaped knowledge production and interpretation. Given that the first author organized and led all focus groups, their positionality likely influenced the research process. The alignment between the first author's and many participants' identities may have facilitated rapport, encouraging participants to share more openly and enabling the collection of richer data. As a group, the four authors represent diverse identities, and all have shared research interests in evidence‐based practice and culturally attuned systemic therapies.

## 5 Results

Participants shared complex and nuanced feedback regarding their observations of the videos as well as their own experiences of EFCT, from which the unanticipated theme of othering emerged. All participants noted that they specifically sought out a therapist who had experience working with the LGBTQ+ communities. However, the degree to which participants desired their LGBTQ+ identities integrated into EFCT varied, leading some participants feeling othered. Indeed, the ways in which identity was talked about—or not talked about—both in therapy and in the training videos were experienced in complex ways. Three themes emerged from negative case analysis related this experience of othering, including (1) understanding versus inflating identity, (2) educating the therapist, and (3) deepening discussions around identity.

### 5.1 Theme 1: Understanding Versus Inflating Identity

All participants agreed that therapists *must* have experience in working with LGBTQ+ communities; it was important for participants that their therapist understood what it meant to non‐heterosexual and/or not cisgender. However, some cisgender participants explained that, if the therapist were to discuss their sexual identity, they would feel othered—they would experience their identity as inflated, thereby creating a comparison between straight/cisgender and LGBTQ+ relationships, meaning that their relationship was not “on the same plain as any other relationship” (P15). By creating this comparison, participants reported feeling as though therapists did not know about LGBTQ+ relationships and/or that there was an inherent and problematic difference between their relationships and heterosexual/cisgender relationships.

Indeed, several participants noted that if the therapist were to discuss their sexual identity, it would mean that their relationship was abnormal. P2 explained that therapy was not a space for gender or sexuality to be “called out” because they “just wanted to be the same as everyone else.” Therefore, the absence of discussion of sexuality meant that they were “basically just like everyone else” (P11). By discussing identity, it would make participants “feel different” (P11). This directly tied into feeling safe in the therapeutic relationship, as P8 explains: “[our sexuality] was a non‐issue and that's how I felt safe, because it was nothing.” P7 summarized this, stating: “I think it's really [never] been mentioned, which feels like total acceptance.”

Additionally, several of the participants voiced that LGBTQ+ identity was irrelevant to their presenting problem. Participants provided several reasons for this: they felt sufficiently secure in their lesbian identity that it did not feel relevant (e.g., P7, P16, P17), their queer identity was fully accepted by their partner (e.g., P27, P28), their presenting problems were experiences unrelated to LGBTQ+ marginalization (e.g., P7, P8, P11, P12, P14, P24), and because navigating interracial relationship challenges took precedence (e.g., P12, P13, P14). This made watching the stage one training tape, in which the therapist openly discusses gay male identity, challenging to view for some participants. For P4, “[the therapist] made their gayness an issue and the issue was safety and vulnerability…. Don't make being gay an issue. If it's an issue…you shouldn't be counseling a gay couple.”

On the other hand, two non‐cisgender participants noticed that the focus on gender identity to the point of excluding the presenting problem and ignoring other parts of a couple's identity also felt othering. P27 explained about their own experience in EFCT: “…this assumption was made around my transness being an issue, especially an issue in my attachment with others. In my individual session [our therapist] focused entirely on my trans identity.” (P27). P27's partner (P28), who identified as non‐binary, further explained how this therapist's focus on trans identity felt othering: “It was really upsetting that [our therapist] didn't even acknowledge my [non‐binary] identity. And neither of us felt like [our individual sessions] were related to our relationship.” The focus on identity—on certain identities—as opposed to what clients find relevant amplified the feelings of being othered and amplified experiences of marginalization.

### 5.2 Theme 2: Educating Therapists

Participants often sought therapists with LGBTQ+ experience so they would not have to educate the therapist about LGBTQ+ identities and experiences; as P20 stated, having a therapist familiar with the lived experiences of being LGBTQ+ meant they did not have to “do all the damned explainings.” Therefore, being responsible for teaching therapists about LGBTQ+ communities was perceived as something marginalized individuals were routinely tasked with doing and a primary method of feeling othered. P35 explained: “I'm paying to educate [the therapist] about a thing that is relatively reasonable to expect that [they] would know. That's not something I think that we would expect of non‐minority folks.” The need to educate the therapist about the LGBTQ+ communities also caused participants to discuss the lack of safety inherent in therapists learning about their identities during therapy. For example, P24 stated: “I wouldn't feel as supported…having to explain what it's like to be queer.” P3 succinctly summarized: “I'm not here to teach you how to be gay.”

Several participants reported that it was important for the therapist to learn—but also ensure that the focus remained on the client's experience rather than the therapist learning. This was especially clear for participants who noticed that the therapist in the training video repeated multiple times that a particularly common experience of being gay man (being hyper independent as so to avoid being thought of as feminine) had not occurred to her. The focus of therapy shifted from being about the client's relationship to the therapist's own experience of grasping new information. P31 noted: “I think what's really othering is to have someone respond to some aspect of your identity or experience with a lot of shock.” This underscored that participants' sexuality and, therefore, inherent parts of themselves were other, alien, foreign—and that they could not expect safety from therapists who othered them. This observation of the lack of safety angered some participants, as P14 explained:

The [therapist's] response rubbed me the wrong way… [the client in the training video] brought that in that he was a young gay boy, which had different implications and any emotionality was ascribed to femininity. Which had a very negative connotation. And when the therapist said ‘I never thought about that’…I was like…you should know that. [I know] this was filmed in the early 2000s and education for therapists and queer identity just wasn't there. It still isn't there…but that never occurred to you?

On the other hand, some participants noted that it was important for the therapist to be open to learning from their clients. P15, P16, P17, P21, and P22 all mentioned that their therapist's genuine curiosity about their lived experiences was meaningful and engendered safety. This duality—the notion that it is important for therapists to learn, and it is also important for therapists to not voice that learning to their clients, especially when related to sexual and/or gender identity—underscores the complexity of how and when LGBTQ+ clients felt othered.

### 5.3 Theme 3: Discussing and Deepening Identity

Participants also noted that in some circumstances, it helped for therapists to openly discuss LGBTQ+ identities. P16 and P17 reported that their therapist asked about their pronouns and broached her identity at the start of therapy, leading to a sense of comfort and safety. Moreover, all participants who identified as non‐White reported that discussing how race and ethnicity impacted their relationship dynamics was essential to ensuring safety within the therapeutic relationship. All participants who named that discussing identity was important emphasized that identity should not be broached at a surface level, but rather a deeper discussion of identity and its implications was needed.

Part of deepening the discussion of identity meant that the therapist needed to move beyond stereotypes. Indeed, when observing the training video, P11 noted how important therapist word choice is when discussing an identity that the therapist does not share with their clients: “… [the therapist] didn't even say *it's like* [sic] you're a stranger in a strange land. She said: ‘*you are* a stranger in a strange land.’ I thought, you can't really speak to a queer person's experience if you're not queer.” The emphasis on language, on how the therapist makes statements about identity, stresses the importance of asking questions and working from a not knowing position, in which the clients are the experts.

The importance of asking questions, of using questions to dive deeper was emphasized when participants pointed out moments the expert therapist could have deepened the discussion of identity in the training videos: “…they were talking about gender roles, and [the therapist] is talking about men in general. There [were] so many opportunities to reflect and put their sexuality and their relationship within the frame of attachment” (P34). P1 noted that the expert therapist did not deepen the discussion related to coming out—which prevented the therapist from both understanding the client in a deeper way and prevented the couple from discussing how they created sufficient safety in their relationship for one member to come out at work for the first time. Several participants noted this was a crucial miss, as coming out can be a rite of passage for LGBTQ+ individuals, fraught with both joy and fear.

While the therapist in the training video did discuss the duality of “external oppression” and the subsequent experience of internal oppression, there was “some depth missing in how [the therapist] responded to those two elements” (P31). P30 elaborated:

…initially, I thought what a powerful thing that she chose to bring up the topic of the messaging of masculinity…she's naming this other force the same way she's naming the internal forces and the attachment forces at play. And then as soon as it got to the moment where it was outside of what she could comprehend…reductivist is the perfect word—like trying to bring this back down to a place where she understands it and it's purely an individualistic choice and an individualistic dynamic.

Indeed, one of the gay men in the stage one training tape mentioned that he could “potentially be destroyed” for coming out. P11 noted this and observed: “that was such an important, emotionally big thing to say. And [the expert therapist] repeated it, but she didn't go anywhere with it.” The lack of depth, the lack of deepening, resulted in feeling othered. Moreover, the lack of ability to hold the complexities of intersectionality in relationship was not only observed in the training videos but also felt in participants' own experiences in EFCT. P35, who identified as genderfluid, explained that they could not bring their entire self and all the nuances of their relationship to therapy:

…in the car on the way home, we talked about…do we want to just not try and have [our therapist] understand my gender? Like there were just so many layers that we just [let go of]…. We'll just let her treat us as a cis/het couple and we'll see if it works that way…. I think we got more from the following sessions after we let [our] nuances go.

The lack of deepening identity, the inability to hold complexity, and the normalization of treating clients “the same way as every hetero couple” (P21), left some participants feeling alien, abnormal, and othered, while leaving others longing for their identities to not overshadow the purpose of therapy.

## 6 Discussion

The current study documents the unanticipated finding of othering experienced by members of the LGBTQ+ communities in EFCT using negative case analysis. Participants reported that why, when, and how LGBTQ+ identity was discussed—or not discussed—impacted their experience of both therapy and observing the training videos. Specifically, participants described that the discussion of identity could indicate that the therapist was conflating their sexual identity with their presenting problem, that the way that identity was discussed could indicate clients needed to spend time educating the therapist about common experiences within the LGBTQ+ communities, and that, if the therapist was going to discuss identity, the conversation should be focused on deepening understanding of the client's lived experiences. These findings suggest that the discussion of LGBTQ+ identity is nuanced and intersectional, as there is a diversity of thought around what each individual (and relationship) preferred.

Participant preferences regarding the discussion of their sexual identity highlight essential aspects of culturally responsive therapy, including the value of open dialogue to determine client preferences for session focus and the importance of aligning treatment with client worldview. Client preference accommodation results in less dropout and improved outcomes (Swift et al. 2018) and clients prefer therapists who use open discussion to adapt therapy to account for their unique needs (Di Malta et al. 2025). Moreover, using therapy models, methods, and interventions that align with client values, beliefs, and assumptions (i.e., worldview) is both a core tenant of cultural humility (Mosher et al. 2017) and can engender culturally safe care (Wright et al. 2021).

Research supports the use of broaching in therapy (Chang and Berk 2009; Sanders Thompson and Alexander 2006; Zhang and Burkhard 2008; Zhang and McCoy 2008). Broaching refers to a consistent openness and commitment to exploring issues of diversity, culture, identity, power, privilege, and oppression with clients (Day‐Vines et al. 2020; Pettyjohn et al. 2020). Broaching involves several different dimensions, including intracounseling (i.e., the acknowledgment of the similarities and differences between counselor and client), intraindividual (i.e., the acknowledgment of the intersectional nature of identity), intracultural (i.e., the acknowledgment of potential concerns arising in the therapist's ingroup), and intercultural (i.e., the therapist's acknowledgement of the aspects of oppression impacting client distress [Day‐Vines et al. 2020]). Broaching outcome research supports greater provider credibility, richer client self‐disclosure, greater likelihood to return, enhanced working alliance, and superior therapeutic outcomes (Chang and Berk 2009; Sanders Thompson and Alexander 2006; Zhang and McCoy 2008). Therapists who broach are viewed as more culturally competent by their clients (Allen 2024), especially LGBTQ+ clients (Quiñones et al. 2015).

There is no correct answer to navigating the nuance of client preference regarding identity discussion and therapist identity disclosure; this discussion is not one size fits all. Although therapists who broach identity are seen as more culturally competent and extant research supports broaching behavior (Chang and Berk 2009; Sanders Thompson and Alexander 2006; Zhang and Burkhard 2008; Zhang and McCoy 2008), therapists also risk harming the therapeutic relationship. For example, the therapeutic relationship can be damaged when therapists discuss race with clients who adhere to colorblind attitudes (Allen 2024) and therapists who broach disability status risk increased client distress due to clients feeling judged by the therapist (Anderson 2024). Therefore, we recommend speaking both from a place of research when naming why the therapists believe it is important to discuss client and therapist identities and also moving slowly, using consent, checking in with clients regarding what they feel comfortable discussing, and following the client's lead.

Research‐based models such as an intersectionality framework (Crenshaw 1989) enable therapists to consider how multiple intersecting identities may influence clients' lived experiences, while engaging clients in their own perspectives regarding issues of privilege and disadvantages that uniquely impact their daily lives. The ADDRESSING model (Hays 2024) and others alike may complement such frameworks, allowing therapists to assess and acknowledge structural experiences that perpetuate bias and discrimination, while remaining proximal to couples' lived realities by allowing them to share *if* and *how* their health as individuals and a couple system may be impacted by marginalization. These frameworks not only enhance awareness, knowledge, and humility around experiences that differ from our own, but also allow for self‐examination around common assumptions and behaviors that may intentionally or unintentionally perpetuate microaggressions in therapy and ultimately compromise client outcomes (Davis et al. 2016).

An adage of therapy is to “meet clients where they are.” These findings indicate that it is important for therapists to meet their clients in their stage of identity development. Identity refers to an individual's continuous sense of self which is defined by their individual physical, psychological, and interpersonal characteristics, as well as their affiliations and social roles which are derived from the feeling that one's memories, beliefs, and values belong to the self (American Psychological Association 2022). There are various identity development models (e.g., Bishop et al. 2020) that outline how members of the LGBTQ+ communities understand and integrate their non‐heterosexual/non‐cisgender identity. For example, one of the most detailed models of lesbian, gay, and bisexual identity development was described by Cass (1979).

Cass (1979) asserts identity is acquired through developmental processes and behavior change and stability lies in the interaction between an individual and their environment. Individuals can possess both public and private identities and with increasing development, these aspects of identity are integrated and become more congruent. This model includes six stages from confusion (i.e., a recognition that non‐heterosexual information is personally relevant and experiences of incongruency in their view of self) through to a third stage noted as tolerance (i.e., increasing contact with others who identify as non‐heterosexual), and finally a sixth stage noted as synthesis (i.e., allowance for flexibly and ideographically evaluating both LGBTQ+ and heterosexual individuals as well as acceptance of a non‐heterosexual identity as *one* part of the myriad aspects of self [Cass 1979]). Identity foreclosure may occur if both self and others' reactions to identity recognition and/or disclosure are perceived as negative.

Despite early widespread acceptance of Cass' model, important critiques have emerged questioning a linear stage approach to identity development (e.g., Horowitz and Newcomb 2002). These critiques include the limits of a unidirectional endpoint development model which does not allow for conceptualizing identities in more complex schemas that include continuous evolving identities that are iterative and shaped by developmental, sociocultural, interpersonal, and historical contexts (D'Augelli 1994; Diamond 2006). Another critique is that Cass' and other early models of development were primarily cultivated from small, community samples consisting primarily of White gay male adults. Bishop et al. (2020), for example, found the value of intersectional and life‐course approaches to sexual identity development was important noting substantial variation in the developmental timing and pacing of milestones among lesbian, gay, and bisexual participants. While completing a system review of the longitudinal research evidence surrounding gender identity development, Fisher et al. (2025) found that that distinct developmental patterns can be observed when using different measure and constructs of gender identity. From an attachment perspective, the parts of self consisting of sexual and/or gender identity may be cut off or suppressed based on sociocultural context and lived experience. It may be unsafe for clients to explore these parts of themselves in therapy, and they may need consistent and subtle broaching by the therapist to model how to safely discuss their identities. It is also possible several of the participants who noted not desiring identity to be integrated into therapy may have foreclosed on further identity development.

Considering identity as embedded in sociocultural contexts and social dimensions (Margi and McQueen 2023) allows one to consider how relationships are impacted by the foreclosure of identity and describe potential reasons for engaging identity as part of relational therapeutic work. For example, while adaptive, identity concealment (i.e., the tendency to maintain privacy regarding one's sexual orientation and same‐sex romantic relationships (Mohr and Kendra 2011]) is negatively associated with relationship satisfaction (Guschlbauer et al. 2019; Pepping et al. 2019) and positively associated with psychological distress (McIntyre et al. 2021; Riggle et al. 2017). Therefore, increasing relationship satisfaction and decreasing distress inherently involves identity integration for members of the LGBTQ+ communities, providing additional support for EFCT therapists integration of identity discussion.

Additionally, the experiences of these participants underscore the importance of therapist use of the MCO framework and, specifically, cultural humility. Indeed, therapists who incorporate cultural humility into their work are motivated to learn from others over the course of their life, critically examine their own cultural awareness, emphasize interpersonal respect, focus on developing collaborative partnerships with their clients that address power imbalances, and take an other‐oriented stance which allows them to accept new cultural information (Mosher et al. 2017). The participants in this study named that the therapist in the training videos did not always practice cultural humility, and this was especially salient in how the therapist examined their own cultural awareness and accepted new cultural information. These experiences highlight the importance of being both fully present with clients when they share cultural information that is new to us as therapists and also how therapists process that cultural information in the moment. This necessitates in the moment reflection regarding how the therapist responds to the new cultural information both verbally and non‐verbally.

### 6.1 Limitations

While this study provides important insights into how LGBTQ+ clients experience othering, this study cannot possibly describe all the possible ways in which this could occur. This may be especially true for participants navigating intersectional and other marginalized identities, such as race, ethnicity, class, and ability status, among others. Indeed, most of this sample identified as White and over the age of 30, limiting generalizability to members of LGBTQ+ communities that are younger and are people of color. A further limitation of this study was that the first two focus groups were unable to see the videos due to technology challenges. While they could hear the audio, it is possible that they may have missed information that could only be received by viewing the tape and/or their attention may have wavered more. Participants also made several assumptive statements about what would fit best for certain groups under the LGBTQ+ umbrella (e.g., gay men, lesbian women). Therefore, it should be noted that participants can only speak to their own lived experiences despite their more encompassing statements. Finally, participants spoke from their own experience in therapy. As we did not interview or assess any of the EFCT therapists that referred their clients to the study, we cannot be sure if they are reacting to a ‘pure’ EFCT or an adapted version.

## 7 Implications for EFCT Training and Practice

The findings from this study support a shift in how EFCT therapists engage with some EFT microskills (e.g., empathic reflections, validation, evocative responding, heightening, empathic conjecture, and choreographing engaged encounters) to avoid othering. For example, participants highlighted the importance of asking questions rather than relying on reflection and summarization. The use of questions allows clients to make a choice regarding their experience; therefore, a shift toward *evocative questions* rather than *evocative reflections* when working with clients holding marginalized identities may be warranted. Indeed, participants noted the importance and deep impact of therapist curiosity on increasing safety.

One indispensable aspect of the implementation of both cultural humility and broaching is therapist training. Quality training can improve therapist comfort with broaching (Askren 2022) and the use of the MCO framework (Davis et al. 2018), especially in the context of supervision (Winkeljohn Black et al. 2025). We, therefore, highly recommend EFCT therapists seek out trainings and supervision focused on both broaching and the MCO framework (Davis et al. 2018). In addition to training on cultural humility models, findings from this study also suggest that EFCT therapists may benefit from increased awareness of complementary research‐based models (e.g., intersectionality, ADDRESSING) to remain cognizant of clients' potential experiences when living with multiple intersecting identities that are marginalized. It is essential that EFCT therapists are comfortable discussing their own and their client identities, practice cultural humility, take advantage of cultural opportunities, and create sufficient safety to allow for repair when ruptures related to othering occur.

Participants in this study believe othering can be avoided by therapists intentionally bearing witness to their client's experiences of marginalization and oppression. By approaching client experiences with cultural humility and avoiding the imposition of therapist values, LGBTQ+ clients are less likely to be made to feel othered. Finally, therapist competencies and guidelines often mention the integration of identity development into the therapeutic process (e.g., Ratts et al. 2016). Given the link between attachment and identity development, it is important for EFCT therapists to be familiar with these models and integrate them into their therapeutic work with LGBTQ+ clients.

The findings from this study strongly support the importance of therapists intentionally integrating client identity within a multicultural framework. Beginning therapy with minimal discussion of identity may help therapists remain responsive to each client's preference regarding whether their sexual and/or gender identities should be incorporated into treatment. When identity is addressed, therapists should engage in exploration that is both broad and in‐depth to ensure it is meaningful and relevant to the client's experience.

## Data Availability

The data that support the findings of this study are available on request from the corresponding author. The data are not publicly available due to privacy or ethical restrictions.
